# Child maltreatment victimization by type in relation to criminal recidivism in juvenile offenders

**DOI:** 10.1186/s12888-016-0731-y

**Published:** 2016-02-05

**Authors:** Claudia E. van der Put, Corine de Ruiter

**Affiliations:** Forensic Child and Youth Care Sciences, University of Amsterdam, P.O. Box 94208, 1090 GE Amsterdam, The Netherlands; Faculty of Psychology and Neuroscience, Section Forensic Psychology, Maastricht University, P.O. Box 616, 6200 MD Maastricht, The Netherlands

**Keywords:** Types of child maltreatment, Criminal recidivism, Juvenile offenders, Gender differences, Risk factors

## Abstract

**Background:**

This study aimed to examine the relation between different types of child abuse victimization and criminal recidivism among juvenile offenders.

**Method:**

Secondary analyses were conducted on data collected with the Washington State Juvenile Court Assessment and general recidivism. The sample consisted of female (*n* = 3502) and male (*n* = 10,111) juvenile offenders.

**Results:**

For male juvenile offenders, neglect and physical abuse victimization were significantly but rather weakly associated with both general and violent recidivism. For female juvenile offenders, neglect and physical abuse were weakly associated with general recidivism, but not with violent recidivism. Sexual abuse was not related to either general or violent recidivism in both male and female juvenile offenders. Most associations between dynamic (treatable) risk domains and recidivism were stronger in male juvenile offenders than in female juvenile offenders. In addition, most risk domains were more strongly related to general recidivism than to violent felony recidivism. For male juvenile offenders, neglect victimization was uniquely related to general recidivism whereas physical abuse victimization was uniquely related to violent recidivism, over and above dynamic risk factors for recidivism. For female juvenile offenders none of the maltreatment variables were uniquely related to general or violent felony recidivism.

**Conclusions:**

Childhood experiences of neglect and physical abuse predict reoffending in male juvenile offenders, pointing at a possible need to address these in risk management interventions.

## Background

Although many studies have focused on the association between child maltreatment and later delinquent behavior (e.g., [1–6]), only few studies have examined the relation between child maltreatment and criminal *recidivism* among delinquent populations. To be able to effectively treat offenders with a history of child maltreatment, more knowledge is needed on the relation between child maltreatment and criminal recidivism. Because many of the problems youth face as a consequence of child maltreatment put them at increased risk of becoming delinquent, it is assumed that the relation between child maltreatment victimization and criminal behavior is mediated through several “dynamic” risk factors [7]. Dynamic risk factors for recidivism are social and individual characteristics that increase the likelihood of recidivism and can potentially be changed. Therefore, these risk factors are often the focus of treatment for juvenile offenders [8]. Examples are poor school performance, mental health problems, truancy, antisocial peers and conflicts in the family. The aim of this study was to examine: (1) the relation between specific victimization types and recidivism and (2) the unique contribution of specific victimization types to the prediction of recidivism over and above dynamic risk factors for recidivism. If there is a unique contribution of child maltreatment to recidivism, treatment should probably not only focus on these dynamic risk factors for recidivism but also on the problems associated with trauma experienced as a child.

Child maltreatment involves a wide range of harmful behaviors directed towards children (i.e., physical abuse, sexual abuse and neglect), which may have different effects on criminal recidivism. Only a few studies examined the association between *specific* types of child maltreatment victimization and recidivism, because in a substantial proportion of cases, various types of maltreatment co-occur [9, 10], which makes it difficult to isolate the effects of specific types of abuse. In addition, due to small sample sizes, the different types of child maltreatment are often combined in analyses. Dembo and colleagues [11] examined the relation between specific types of child maltreatment victimization and recidivism and found that recidivism was more strongly predicted by neglect than by both physical and sexual abuse. Kingree, Phan, and Thompson [12] examined the effects of physical abuse, sexual abuse, emotional abuse, physical neglect and emotional neglect on recidivism, and found only the last two maltreatment types to be significantly associated with recidivism. Ryan, Williams and Courtney [13] examined the relation between neglect and recidivism and made a distinction between adolescent offenders with a history of child neglect and adolescent offenders with an ongoing case of neglect. The study showed that only ongoing neglect during adolescent was significantly related to recidivism after controlling for a wide range of family, peer, academic, mental health, and substance abuse covariates [13].

The relation between specific types of maltreatment and *delinquency* has been studied more extensively. Some studies provided evidence that specific victimization types predict a specific type of offending behavior (e.g., [9, 14–18]. For example, victims of physical abuse more often show violent offending behavior [15] and victims of sexual abuse more often commit sexual offenses [14]. Other studies suggest that the propensity towards crime in general depends on the type of abuse experienced [19, 20]. There are indications that victims of neglect and physical abuse are at greatest risk of delinquency, whereas sexual abuse victims are no more at risk of offending than juveniles who were not a victim of maltreatment [19, 20]. Trickett and McBride-Chang [21] reviewed the literature on the impact of different types of child maltreatment (physical abuse, sexual abuse and neglect) and found that physically abused juveniles showed more externalizing problems, compared to sexually abused juveniles who showed more internalizing problems. However, they suggested that these differences may in fact be gender-differences because females predominate in the samples of sexual abuse victimization studies and they have a stronger tendency to develop internalizing problems [22] relative to men.

Other researchers also suggested gender differences in the relation between child maltreatment and delinquent behavior: females are considered more likely to internalize their reactions whereas males are more likely to externalize their reactions to child maltreatment [23–25]. These differences in reactions may be one of the explanations for the finding of Topitzes and colleagues [26] that child maltreatment predicted juvenile delinquency in males, but not in females. However, Topitzes and colleagues [26] found that child maltreatment predict adult crime for both genders, which suggests that the effects of child maltreatment on delinquent behavior may be delayed in girls. In a review of findings from the child welfare and juvenile delinquency literature, Bender [7] proposed a direct effect of child maltreatment on delinquency in boys but not in girls. In an earlier study, we found stronger associations between child maltreatment victimization and offending behavior in male compared to female juvenile offenders [27]. Compared to female juvenile offenders, sexual abuse victimization was more strongly related to sexual offending, and neglect was more strongly related to violent offending, in male juvenile offenders [27]. Other researchers, however, have suggested that the consequences of child maltreatment play a greater role in the development of delinquent behavior in females than in males [28–30]. Because of the mixed findings regarding gender differences in the relation between maltreatment and delinquent behavior, the second aim of the present study was to examine gender differences in the relation between specific victimization types and recidivism and in the *unique contribution* of specific victimization types to the prediction of recidivism.

## Method

### Sample

For this study, secondary data from the Washington State Juvenile Court Assessment (WSJCA) validation study were used [31]. This dataset consists of 13,613 American juvenile offenders (3502 girls and 10,111 boys), aged 12 to 18, who were convicted by a juvenile court and for whom the WSJCA was completed. The WSJCA is a screening and risk assessment instrument, which comprises two parts: pre-screen and full assessment (see Instrument section). The pre-screen is administered to all youth on probation with the aim to indicate whether a youth is at low, moderate or high risk for reoffending. The full assessment is required for youth who are assessed as moderate or high risk on the pre-screen with the aim to identify a youth’s risk and protective factor profile to guide intervention targeting desistance from crime and rehabilitation. The current sample only included those offenders for whom the full assessment was performed, which indicates that the participants had a medium to high recidivism risk on the pre-screen.

### Instruments and procedure

#### Washington State Juvenile Court Assessment (WSJCA)

The WSJCA is a screening and risk assessment instrument, developed in Washington State [31, 32]. The WSJCA maps out the most important risk and protective factors for *criminal recidivism* on a large number of domains. The development of the instrument was based on a review of the following types of research: recidivism prediction literature and validity studies of risk assessment instruments, for example: the Wisconsin Risk Scale [33] and the Youth Level of Service Case Management Inventory [34], risk and protective factor research, resiliency research and research on effective juvenile delinquency treatment programs (see [31]). The selection of domains and items took place on the basis of this review and was subsequently modified, based on feedback from an international panel of experts (see [31]).

Probation officers complete the WSJCA during the intake, on the basis of information from a structured motivational interview with the youth and his/her family. Probation officers are trained in conducting the assessment by certified trainers. This training includes reviewing video-taped interviews and the resulting assessment to ensure the probation officer has mastered the assessment skills. There is a manual available for the WSJCA and quality assurance is an important part of the assessment structure and organization in Washington State [34].

The WSJCA measures both static (historical) and dynamic (current) factors. In the present study we only examined *dynamic* risk factors because these factors are used to guide the rehabilitative effort. The dynamic factors were measured over a period of six months prior to the assessment, so the dynamic risk factors were present at the time of the assessment or shortly before (maximum six months). All questions were asked to both the youth and the parents. The items concerning schools were checked with the schools the juveniles were attending. If conflicting answers were given by the youth and his parents, the probation officer made an estimation of the accuracy of the answers and the most appropriate response, based on his or her clinical experience and knowledge or by collecting additional information, for example by consulting school or other professionals who know the youth.

The dynamic risk factors examined in the present study were risk factors in the following domains: (a) *School*: severe behavior problems, poor academic performance (some Ds and mostly Fs), truancy, poor relationship with teachers, not interested or involved in school activities, not likely to graduate, (b) *Alcohol/drugs:* alcohol and/or drugs causing family conflict and/or disrupting education and/or causing health problems and/or interfering with keeping prosocial friends and/or drugs contributing to criminal behavior, (c) *Relationships:* no positive adult non-family relationships, no prosocial community ties, antisocial friends or gang membership, romantically involved with an antisocial person, admires or emulates antisocial peers, rarely resists antisocial peer influence, (d) *Family:* low family income (annual income under $15,000), poor relationship with parents, serious conflicts in the family, inadequate parental supervision, youth consistently disobeys family, no family support network, poor parental punishment and parental reward (inconsistently or consistently insufficient), (e) *Aggression:* low frustration tolerance, believes verbal aggression is often appropriate to solve a conflict, believes physical aggression is sometimes or often appropriate to solve a conflict, hostile interpretation of other’s behavior/intentions, (f) *Attitude:* low aspirations, impulsiveness, no control over anti-social behavior, no empathy, no respect for others’ property, no respect for authority figures, no respect for rules/social conventions, does not accept responsibility for behavior, does not think he or she can comply with measures, (g) *Skills*: poor consequential thinking, poor goal setting, poor problem-solving behavior, poor situational perception, problems in dealing with others, lacks skills in dealing with difficult situations, lack of skills in dealing with feelings/emotions, problems in controlling internal and or external triggers, lacks techniques to control impulsive behavior, lacks alternatives to aggression, (h) *Mental health problems*: psychotic, mood, anxiety, personality and adjustment disorders. For each domain, a total risk score was calculated by adding the scores of the individual risk factors within that domain.

Physical abuse, sexual abuse and neglect were operationalized following Child Protective Services (CPS) definitions in the US [32]. Physical abuse included any non-accidental physical injury, such as bruises, burns, fractures, bites, or internal injuries. Sexual abuse included acts such as indecent liberties, communication with a minor for immoral purposes, sexual exploitation of a child, child molestation, sexual misconduct with a minor, rape of a child, and rape. Neglect included negligence or maltreatment (dangerous act) or omission that constitutes a clear and present danger to the child’s health, welfare, and safety, such as: a) failure to provide adequate food, clothing, shelter, emotional nurturing, or health care, b) failure to provide adequate supervision given the child’s level of development, c) an act of abandonment with the intent to forego parental responsibilities despite an ability to do so, d) an act of exploitation, such as requiring the child to be involved in criminal activity, imposing unreasonable work standards, etc., e) an act of reckless endangerment, such as a parent driving under the influence of alcohol or drugs with children present and, f) other dangerous acts. The self-reported information was checked with child protective services, community mental health organizations, and other sources of information. Any history of being a victim of neglect, physical or sexual abuse by a family member that was suspected was included. False reports of abuse or neglect were excluded [32].

The predictive validity of the WSJCA pre-screen has been tested in three studies [31, 35, 36]. In the first study [31], the Area Under the receiver-operating-characteristics Curve (AUC) was .64 and in the subsequent studies the AUC was .63. In a meta-analysis of the predictive validity of risk assessment instruments for juveniles, it was shown that the AUCs varied from .53–.78, with an average AUC of .64 [37]. The AUC found for the WSJCA pre-screen is therefore comparable to the average AUC of juvenile justice risk assessment instruments. The validity of full assessment was examined in a study of Barnoski [31] in which both the domain score structure as well as the predictive validity of the domain scores was examined. The factor analysis of the 29 domain scores confirmed the relative independence of the assessment domains. Few domain scores loaded on more than one factors. The factor analyses of the items within each domain illustrated that most domains were multifaceted, measuring more than a single concept. Two domains, free time and alcohol/drug use, were one-dimensional. The risk and protective domain scores were significantly associated with felony recidivism (convictions). The risk domain scores were more closely associated with recidivism than the protective domain scores.

#### Outcome measure

General recidivism was defined as the occurrence of one or multiple new convictions for any kind of offense within 18 months after completing the WSJCA. Violent felony recidivism was defined as the occurrence of one or multiple new convictions for a violent felony offense within 18 months after completing the WSJCA. To adequately measure 18-months recidivism, a period of 30 months was needed for gathering the information: a 18-months re-offending follow-up period and another 12-months period to allow for any re-offenses to be adjudicated [38]. Recidivism was treated as a dichotomous variable (yes or no conviction for a new offense during follow-up).

### Analyses

First, we examined differences in the prevalence of child maltreatment and criminal recidivism between male and female juvenile offenders by using chi-square analysis. Second, we examined bivariate associations between different types of child maltreatment and recidivism and between dynamic risk factors in different domains and recidivism by using Pearson correlation coefficients, separately for general and violent felony recidivism and for male and female juvenile offenders. Fisher’s z tests were used to examine whether the correlations differed significantly between male and female juvenile offenders. Second, we performed multivariate logistic regression analyses to examine the unique contribution of child maltreatment to the prediction of criminal recidivism, over and above dynamic risk factors for recidivism, separately for male and female juvenile offenders and separately for general and violent felony recidivism.

### Ethical approval

Formal Institutional Review Board (IRB) approval to conduct this study was not required, as this study involved secondary data analysis on de-identified data, which does not pose harm to the subjects and therefore does not necessitate IRB regulation. Accordingly, this study was ethically conducted based on the rules maintained by the Faculty Ethics Review Board (FMG-UvA) of the University of Amsterdam, The Netherlands. The Washington State Institute for Public Policy has given permission to use the data for this study.

## Results

Table 1 presents the percentages of sexual abuse, physical abuse and neglect and recidivism rates, for male and female juvenile offenders separately. All forms of abuse (sexual, physical abuse, and neglect) were more often present in female than in male juvenile offenders. General recidivism and violent felony recidivism were both higher in male juvenile offenders than in female juvenile offenders.

**Table 1:** Prevalence of child maltreatment and criminal recidivism rates separately for male and female juvenile offenders

	Male juvenile offenders	Female juvenile offenders	*χ* ^*2*^ *(1)*
	(*n* = 10,111)	(*n* = 3502)	
Victim of sexual abuse	8.0 %	33.2 %	1336.78^*^
Victim of physical abuse	23.6 %	36.0 %	203.16^*^
Victim of neglect	20.8 %	29.4 %	108.77^*^
General criminal recidivism	46.6 %	34.0 %	167.97^*^
Violent felony recidivism	8.5 %	3.2 %	110.57^*^

*Note*: ^*^ *p* < .001

Table 2 shows the correlation coefficients between the different child maltreatment victimization types and recidivism and between the dynamic risk factors and recidivism, separately for male and female juvenile offenders and for general and violent felony recidivism. For male juvenile offenders, neglect and physical abuse victimization were significantly but rather weakly associated with both general and violent recidivism. For female juvenile offenders, neglect and physical abuse were weakly associated with general recidivism, but not with violent recidivism. Sexual abuse was not related to either general or violent recidivism in both male and female juvenile offenders.

**Table 2:** Correlations between different forms of child maltreatment and recidivism and between dynamic risk factors and recidivism separately for male and female juvenile offenders

	General Criminal Recidivism			Violent Felony Recidivism		
	Male juvenile offenders	Female juvenile offenders	*Z*	Male juvenile offenders	Female juvenile offenders	*Z*
	(*n* = 10,111)	(*n* = 3502)		(*n* = 10,111)	(*n* = 3502)	
Victim of:						
Sexual abuse	−.00	.01	.71	−.01	−.00	.20
Physical abuse	.05^***^	.04^*^	.46	.04^**^	.00	1.84
Neglect	.07^***^	.04^*^	1.53	.03^*^	.02	.41
Dynamic risk factors in the domains:						
School	.12^***^	.08^***^	2.06^*^	.04^***^	.04^*^	.00
Relationship	.20^***^	.14^***^	3.15^**^	.12^***^	.04^*^	4.11^***^
Family	.15^***^	.07^***^	4.03^***^	.08^***^	.04^*^	2.05^*^
Alcohol/drugs abuse	.12^***^	.04^*^	4.11^***^	.05^***^	.01	2.04^*^
Skills	.15^***^	.14^***^	.52	.08^***^	.07^***^	.51
Attitude	.20^***^	.15^***^	2.63^**^	.11^***^	.07^***^	2.06^*^
Aggression	.17^***^	.16^***^	.52	.09^***^	.09^***^	.00
MH problems	.04^***^	.06^**^	−1.02	.02^*^	.04^*^	−1.02

*Note*: ^*^ *p* < .05, ^**^ *p* < .01, ^***^ *p* < .001; MH = Mental Health

In both male and female juvenile offenders, risk factors in the domains of school, relationships, family, alcohol/drugs abuse, skills, attitude, aggression and mental health problems were all related to both general and violent felony recidivism, with the exception of alcohol/drugs abuse, which was not related to violent felony recidivism in female juvenile offenders. Most associations between the risk domains and recidivism were stronger in male juvenile offenders than in female juvenile offenders. In addition, most risk domains were more strongly related to general recidivism than to violent felony recidivism, the difference being significant in the domains of school (*z* = 5.73; *p* < .001), relationship (*z* = 5.84; *p* < .001), family (*z* = 5.05; *p* < .001), alcohol/drugs abuse (*z* = 5.01; *p* < .001), skills (*z* = 5.05; *p* < .001), attitude (*z* = 6.56; *p* < .001), aggression (*z* = 5.79; *p* < .001) in male juvenile offenders and the differences being significant in the domains of relationship (*z* = 4.22, *p* < .001), skills (*z* = 2.96; *p* < .01), attitude (*z* = 3.39; *p* < .001), aggression (*z* = 2.98; *p* < .001) in female juvenile offenders.

The unique contribution of childhood victimization to recidivism was examined by performing a hierarchical logistic regression analysis, separately for male and female juvenile offenders and for general and violent felony recidivism. The regression coefficients representing the associations between the dynamic risk factors and *general* recidivism are shown in Table 3. Dynamic risk factors in the domains of school, relationships, family, alcohol/drugs abuse, skills, attitude, aggression and mental health problems were entered at Step 1 and the maltreatment victimization types were entered at Step 2. Only for male offenders, the victimization variables resulted in a significant improvement of the prediction of general recidivism (*χ*^*2*^*(3)* = 12.95, *p* < .01). In male juvenile offenders, risk factors in the school, relationships, family, alcohol/drugs abuse, attitude and aggression domains were uniquely related to general recidivism as well as neglect victimization. For female juvenile offenders, risk factors in the relationships, skills and aggression domains were uniquely related to general recidivism, but the victimization variables did not add significantly to the prediction model.

**Table 3:** Logistic regression coefficients predicting general recidivism from dynamic risk factors and different types of child abuse victimization separately for male and female juvenile offenders

	Male Juvenile Offenders				Female Juvenile Offenders			
	(*n* = 10,111)				(*n* = 3502)			
Gender	B	S.E.	Wald	Exp(B)	B	S.E.	Wald	Exp(B)
Step 1								
School	.03	.01	9.02^**^	1.03	.01	.02	.6	1,01
Relationship	.12	.02	53.98^***^	1.12	.09	.03	12.24^***^	1,10
Family	.02	.01	8.79^**^	1.02	−.00	.01	.09	1,00
Alcohol/drugs abuse	.07	.02	16.26^***^	1.07	−.03	.03	1.16	,97
Skills	.01	.01	2.45	1.01	.04	.02	6.33^*^	1,04
Attitude	.04	.01	10.88^**^	1.04	.02	.02	.61	1.02
Aggression	.06	.02	14.20^***^	1.06	.12	.03	18.33^***^	1.13
MH problems	.06	.05	1.45	1.06	.08	.08	1.04	1.09
∆*χ* ^2^(df)	590.10 (8)^***^				132.07 (8)^***^			
Step 2								
Physical abuse	.06	.06	1.01	1.06	.05	.08	.34	1.05
Sexual abuse	−.17	.11	2.33	.84	−.02	.11	.03	.98
Neglect	.16	.05	8.75^**^	1.17	.07	.08	.62	1.07
Constant	−1.22	.06	466.50^***^	.29	−1.63	.11	209.98^***^	.20
∆*χ* ^2^(df)	12.95 (3)^**^				1.19 (3)			
Total *χ* ^2^(df)	604.05 (11)^***^				133.26 (11)^***^			

*Note*: ^*^ *p* < .05, ^**^ *p* < .01, ^***^ *p* < .001

The regression coefficients representing the associations between the dynamic risk factors and violent felony recidivism are shown in Table 4. Again, only for male offenders, the victimization variables resulted in a significant improvement of the prediction of general recidivism (*χ*^*2*^*(3)* = 9.05, *p* < .05). In male juvenile offenders, only the relationship domain was uniquely related to violent felony recidivism as well as victimization of physical abuse. In female juvenile offenders, only the aggression domain was uniquely related to violent felony recidivism; the victimization variables did not add significantly to the prediction model.

**Table 4:** Logistic regression coefficients predicting violent felony recidivism from dynamic risk factors and different types of child abuse victimization separately for male and female juvenile offenders

	Male Juvenile Offenders				Female Juvenile Offenders			
	(*n* = 10,111)				(*n* = 3502)			
Gender	B	S.E.	Wald	Exp(B)	B	S.E.	Wald	Exp(B)
Step 1								
School	−.01	.01	.73	.99	.01	.04	.05	1.01
Relationship	.17	.03	39.13^***^	1.19	−.02	.07	.04	.99
Family	.01	.01	.87	1.01	−.02	.03	.27	1.02
Alcohol/drugs abuse	.01	.03	.08	1.01	−.03	.08	.15	.97
Skills	.03	.02	3.42	1.03	.08	.05	2.73	1.08
Attitude	.03	.02	2.28	1.03	.03	.06	.23	1.03
Aggression	.05	.03	2.40	1.05	.22	.08	7.45^**^	1.25
MH problems	.08	.09	.87	1.08	.19	.21	.77	1.21
∆*χ* ^2^(df)	173.06 (8)^***^				33.08 (8)^***^			
Step 2								
Physical abuse	.21	.09	5.29^*^	1.23	−.21	.22	.85	.81
Sexual abuse	−.29	.21	1.98	.75	−.13	.30	.20	.88
Neglect	.02	.09	.06	1.02	.14	.22	.43	1.15
Constant	−3.47	.11	938.74^***^	.03	−4.77	.35	186.86^***^	.01
∆*χ* ^2^(df)	9.05 (3)^*^				1.40 (3)			
Total *χ* ^2^(df)	182.10 (11)^***^				34.48 (11)^***^			

*Note*: ^*^ *p* < .05, ^**^ *p* < .01, ^***^ *p* < .001

## Discussion

The aim of the present study was to examine gender differences in the relation between specific child maltreatment victimization types and criminal recidivism and to examine the unique contribution of specific victimization types to the prediction of criminal recidivism, over and above dynamic risk factors for recidivism. For both male and female juvenile offenders, victimization of physical abuse and neglect were significantly related to general recidivism, however, the associations were rather weak. The meta-analysis of Cottle and colleagues [39] also showed that child maltreatment had a weak association with recidivism. The weighted mean effect size (*r* = .11 *p* < .001) found in the meta-analysis was higher than the associations we found in the present study, which may possibly be explained by differences in sample composition. Most of the samples included in the meta-analysis consisted of male juveniles from correctional facilities whereas our sample consisted of both male and female juvenile offenders on probation.

For male juvenile offenders, being a victim of neglect contributed to general criminal recidivism, over and above other dynamic (treatable) risk factors for recidivism, whereas for female juvenile offenders none of the maltreatment victimization variables were uniquely related to general recidivism. Kingree and colleagues [12] also found victimization of neglect to be the only maltreatment type that was uniquely related to recidivism after controlling for demographic (gender, age and ethnicity) and behavioral variables (prior detention, substance use problems, lack of self-restraint and emotional distress). The results of our study contribute to the literature by showing that in male juvenile offenders, victimization of neglect is uniquely related to recidivism after controlling for dynamic (treatable) risk factors in multiple domains (school, family, relationships, alcohol/drugs, attitude, aggression, skills and mental health problems), whereas victimization of neglect is not uniquely related to recidivism in female juvenile offenders.

In addition, only for male offenders, the victimization variables resulted in a significant improvement of the prediction of violent felony recidivism. In male juvenile offenders, being a victim of physical abuse contributed to violent felony criminal recidivism, over and above other dynamic (treatable) risk factors for recidivism, whereas in female juvenile offenders none of the maltreatment victimization variables were uniquely related to violent felony recidivism. A possible explanation for this finding might be that girls are more likely to develop internalizing symptoms as a way of coping whereas males are more likely to develop externalizing problems in response to childhood maltreatment [23–26]. In addition, this finding corresponds to the model proposed by Bender [7] in which there is a direct effect of childhood maltreatment on delinquency in boys but not in girls. To summarize, all types of child maltreatment (sexual abuse, physical abuse and neglect) were more common in female juvenile offenders than in male juvenile offenders, but childhood maltreatment showed a unique association with criminal recidivism in boys, but not in girls.

There are several limitations worth mentioning. First, it is important to realize that the information on maltreatment used in the present study is retrospective in nature, i.e., when juveniles were accused of delinquent behavior, their maltreatment history was identified. This may have affected the results, because their recent justice contact may have affected their perception and interpretation of their childhood experiences. Second, the WSJCA only measured *whether* juvenile offenders were neglected, sexually abused and/or physically abused by a family member and not the duration or severity of the abuse. We therefore could not take these factors into account, while earlier studies showed that more extensive maltreatment was related to higher rates of delinquent behavior (e.g., [4]). Third, the WSCJA was not designed to provide an in-depth examination of risk factors. It is a risk assessment tool that is meant to be used by juvenile justice professionals and clinicians to summarize juveniles’ risks and needs, to classify their overall risk level, and plan treatment and supervision strategies. Fourth, there is no information available regarding the interrater reliability of the WSJCA. However, quality assurance is an important part of the assessment structure and organization in Washington State and probation officers received intensive training to adequately administer and score the WSJCA [31, 32]. Finally, the sample predominantly consisted of moderate- and high-risk youth. Therefore, the results cannot be generalized to juvenile delinquents with low recidivism risk.

## Conclusions

The present study provides relevant information for clinical practice on the relation between specific child maltreatment victimization and recidivism in juvenile offenders. For male juvenile offenders, neglect victimization was uniquely related to general recidivism whereas physical abuse victimization was uniquely related to violent recidivism, over and above dynamic (treatable) risk factors for recidivism. For female juvenile offenders none of the maltreatment variables were uniquely related to general or violent felony recidivism. These results indicate that the treatment of male juvenile offenders should also focus on the direct consequences of physical abuse and neglect. We recommend other treatment modalities addressing the needs of victimized male adolescent offenders in addition to risk-focused treatment (e.g., trauma-focused cognitive behavioral therapy; [40]). For instance, results from two case studies suggest that Prolonged Exposure, a trauma-focused treatment is feasible for treating PTSD in juvenile sex offenders [41]. As the criminal justice system is inherently focused on the offending behavior, underlying mechanisms related to a history of victimization, may well be overlooked. More research is needed to examine the relevance of childhood victimization in the assessment and treatment of juvenile offenders.
